# Construction and Validation of a Novel Ferroptosis-Related lncRNA Signature to Predict Prognosis in Colorectal Cancer Patients

**DOI:** 10.3389/fgene.2021.709329

**Published:** 2021-10-28

**Authors:** Wenqi Zhang, Daoquan Fang, Shuhan Li, Xiaodong Bao, Lei Jiang, Xuecheng Sun

**Affiliations:** ^1^ Central Laboratory, The First Affiliated Hospital of Wenzhou Medical University, Wenzhou, China; ^2^ Department of Gastroenterology, The First Affiliated Hospital of Wenzhou Medical University, Wenzhou, China

**Keywords:** ferroptosis, lncRNAs, colorectal cancer, prognosis, risk signature, nomogram

## Abstract

**Background:** Colorectal cancer (CRC) ranks as the third most common malignancy worldwide but a reliable prognostic biomarker of CRC is still lack. Thus, the purpose of our study was to explore whether ferroptosis - related lncRNAs could predict the prognosis of CRC.

**Methods:** The mRNA expression profiling of colon adenocarcinoma (COAD) and rectum adenocarcinoma (READ) patients in The Cancer Genome Atlas (TCGA) database were downloaded. Univariate Cox and multivariate Cox regression analyses was used to obtain prognostic differently expressed ferroptosis-related lncRNAs (DE-FLs) and a risk signature was developed. Quantitative polymerase chain reaction (q-PCR) was used to validated the different expressions of DE-FLs. The calibration curves, C-index and the receiver operating characteristic (ROC) curves were applied to evaluate the accuracy of nomogram. Gene set enrichment analyses (GSEA) were carried out to explore the biological mechanism between high- and low-risk group and the potential regulated pathway of prognostic DE-FLs in CRC.

**Results:** Forty-nine DE-FLs were identified between CRC and normal tissue. Then, a 4-DE-FLs (AC016027.1, AC099850.3, ELFN1-AS1, and VPS9D1-AS1) prognostic signature model was generated. AC016027.1 was downregulated in CRC tissue; VPS9D1-AS1 and ELFN1-AS1 were upregulated by q-PCR. The model had a better accuracy presenting by 1-, 3-, and 5-years ROC curve (AUC ≥0.6), and identified survival probability (*p* < 0.05) well. Moreover, the risk signature could play as an independent factor of CRC (*p* < 0.05). Further, a nomogram including age, pathologic stage, T stage, and risk score with good prognostic capability (C-index = 0.789) was constructed. In addition, we found biological pathways mainly related to metabolism and apoptosis were down-regulated in high-risk group who with poor outcome. Finally, the functional enrichment showed prognostic DE-FLs may significantly impact bile secretion in CRC.

**Conclusion:** A risk model and nomogram based on ferroptosis-related lncRNAs were created to predict 1-, 3-, and 5-years survival probability of CRC patients. Our data suggested that the prognostic lncRNAs could serve as valuable prognostic marker.

## Introduction

CRC is the third major cause of cancer mortality in industrialized countries, which seriously endangers human health (Siegel et al., 2018). The relevant data of the World Health Organization (WHO) shows that the incidence of CRC has begun to decline in some developed countries, but it still remains increase in the developing world (Zeuner et al., 2014). In new CRC diagnoses, 20% of patients have metastases at presentation and another 25% with localized disease will later develop metastases (Biller and Schrag, 2021), therefore early disease diagnosis is especially critical. Despite the encouraging amelioration in CRC diagnostic and therapeutic methods, a relatively high proportion of CRC patients suffering from poor survival outcomes still exists because of late disease detection and lacking availability of adequate risk-assessment biomarkers (Yang et al., 2021). Thus, it is urgently to identify novel and reliable biomarkers for the individualized diagnosis of CRC, which may potentially improve overall outcome of this disease.

Ferroptosis is considered a nonapoptotic, iron-dependent form of cell death with three hallmarks including oxidation of polyunsaturated fatty acid, redox active iron and lipid peroxide repair loss (Dixon et al., 2012; Jiang X. et al., 2021). Cancer cells are more vulnerable to ferroptosis due to their high demand of iron to support fast proliferation (Hassannia et al., 2019). Recently, evidence is emerging that ferroptosis has a tumor-suppressor effect that could be employed for tumor treatment (Stockwell et al., 2017). For example, BAP1 restrains tumor progression partly through SLC7A11 and ferroptosis (Zhang et al., 2018). Ferroptosis also has great potential to eliminate malignant cells which are resistant to conventional therapy. Shin et al. (2018) reported that inhibition of GPX4 made chemoresistance cancer cells more vulnerable to ferroptosis.

Long non-coding RNAs (lncRNAs) are transcripts with more than 200 nucleotides in length, but being not translated into proteins (Mercer et al., 2009). LncRNA possesses variously functional activity including RNA decay, gene expression and control, RNA splicing, miRNA regulation, protein folding (Chen et al., 2017). Some lncRNAs can also prevent oxidation and thus inhibit ferroptosis as rival endogenous RNAs (Jiang N. et al., 2021). A study found that LINC00336 acted as an oncogene, which bound ELAVL1 using nucleotides 1901-2107 of LINC00336 and the RRM interaction domain and key amino acids of ELAVL1 (aa 101-213), inhibiting ferroptosis (Wang M. et al., 2019). Moreover, recent evidences indicated that identification of lncRNAs could help early disease detection and advance therapy outcomes in CRC patients (Yang et al., 2021). Given their roles in malignant development and disorder of expression in CRC patients, lncRNA is undoubtedly potential to be a reliable diagnostic and prognostic biomarker for CRC.

**FIGURE 1:**
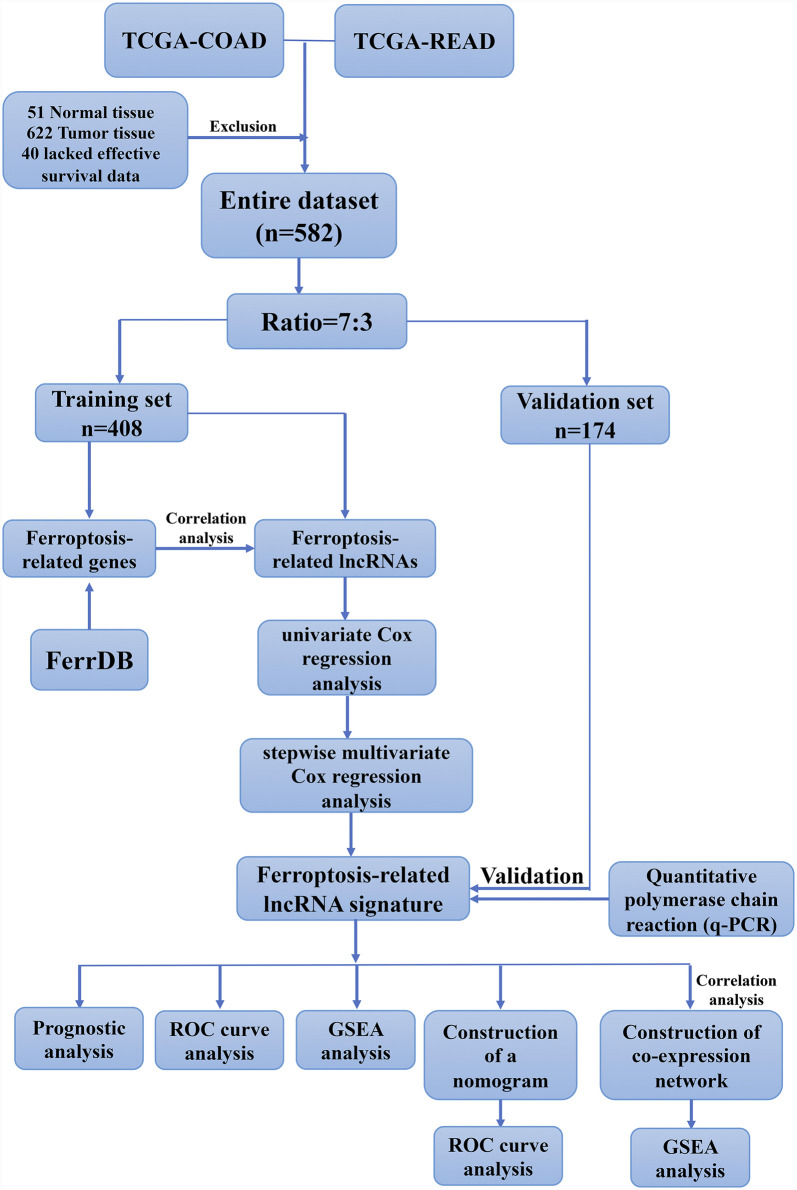
Work flow for the construction of a risk signature in colorectal cancer.

In this study, we hypothesized that ferroptosis-related lncRNAs might be promising prognostic biomarkers for CRC patients. We analyzed the correlation between the expression of ferroptosis-related lncRNAs with survival and the clinicopathological parameter of CRC patients from TCGA database. Moreover, we constructed a prognostic signature based on four ferroptosis-related lncRNAs and assessed its ability to independently and accurately predict the prognosis of CRC patients. The work flow of this study is illustrated in Figure 1.

## Material and Methods

### Acquisition of CRC Data

The level 3 RNA-Sequencing (RNA-Seq) dataset and corresponding clinical information of COAD and READ cancer patients were downloaded from TCGA data portal (https://portal.gdc.cancer.gov/) which was updated at November 9, 2020. There were 673 samples in the dataset, including 622 CRC and 51 normal ones. After excluding the samples without survival information, a total of 582 samples were enrolled in our study and randomly assigned into training and validation set at a ratio of 7:3. In addition, 382 ferroptosis-related genes (FRGs) were obtained from the FerrDb database (http://www.zhounan.org/ferrdb/). GENCODE v22 was used for gene annotation (https://www.gencodegenes.org/human/release_22.html).

### Identification of Differently Expressed Ferroptosis-Related lncRNAs in CRC.

Differently expressed genes (DEGs) and lncRNAs (DLRs) between CRC and normal samples were identified by “limma” package in *R* language. DEGs and DLRs meeting |log2FC| > 1 and *p*_value <0.05 were considered as significantly expressed. The “ggplot2” package were used to construct volcano plot of these DEGs and DLRs. Then we extracted differently expressed ferroptosis-related genes (DE-FGs) from the overlap of FRGs and DEGs, which analyzed by Venn diagram (http://bioinformatics.psb.ugent.be/webtools/Venn/). Pearson analysis were performed to screen the DE-FLs with criterion of |cor| > 0.3 and *p* < 0.05.

### Construction of Prognostic DE-FLs Signature

DE-FLs which significantly associated with CRC prognosis were identified by univariate Cox regression model (cut off <0.2). Then the candidate DE-FLs were entered into a stepwise multivariate Cox regression analysis and constructed a prognostic signature model according to Akaike Information Criterion (AIC). The expression levels of prognostic DE-FLs in normal and CRC tumor samples in TCGA database were checked by Wilcoxon test. And the survival curve of CRC patients based on DE-FLs expression were draw. Further, the risk score of individual patients was established based on the summation of coefficients and expression level of each prognostic DE-FLs according to the following formula:

Risk score =∑(Coefi X Expi) 

Thus, the CRC patients in each set were classified into high- and low-risk group reference the median risk score.

### Evaluation of the Prognostic Signature

To evaluate the valuable of prognostic signature in each set, the differences in patients’ survival between high- and low-risk group were evaluated by Kaplan-Meier curve analyses and log-rank test (*p* < 0.05). Then, the 1-, 3-, and 5-years ROC curves were employed to compare the specificity and sensitivity of the survival prediction based on the prognostic signature *via* “pROC” package. Moreover, the relationship between risk score and clinical characteristics were analyzed by *t-test*.

### Establishment of Nomogram for CRC Prognostic Prediction

To identify independent prognostic factors of CRC, the univariate and multivariate Cox regression analyses were performed to evaluate the risk score and other clinical variables such as age, gender, grade, M stage, T stage, and N stage. The factor with *p* < 0.05 was considered statistically significant. Then, we integrated all of the independent prognostic factors to build a nomogram by “rms” package for inspecting the probability of 1-, 3-, and 5-years overall survival (OS) of the CRC patients. The discrimination and predictive ability of the nomogram in CRC were assessed with calibration curve and a C-index indicate. We also plotted ROC curve and calculated the area under the ROC curve (AUC) values based on total points of nomogram.

### Functional Annotation

Functional enrichment analyses were performed to explore the underlying mechanism of prognostic signature. Firstly, the GSEA software was conducted for Gene Ontology (GO) term and Kyoto Encyclopedia of Genes and Genomes (KEGG) pathways analyses in high- and low-risk group in CRC. Then, the co-expression of the signature DE-FLs and mRNA was assessed with Pearson correlation analyses. The mRNAs in co-expression pairs whose correlation coefficient >0.7 and *p* < 0.0.5 were selected for functional annotation by “clusterProfiler” package. Biological processes were collected from the GSEA (https://www.gsea-msigdb.org/gsea/index.jsp). The *p*. adjust-value below 0.05 was considered significant.

### Quantitative Polymerase Chain Reaction

Thirty pairs of CRC tumor and adjacent tissues were collected from clinical patients at The First Affiliated Hospital of Wenzhou Medical University and preserved in -80 C refrigerator. All patients gave the written informed consent. All assay regimens gained the approval of the Ethics Committees in Clinical Research of the First Affiliated Hospital of Wenzhou Medical University and the corresponding ethical approval code was KY2021-R005. Total RNA was isolated from tissue samples using TRIzol reagent (Invitrogen, Carlsbad, CA). Then PrimeScript™ RT Master Mix kit (TaKaRa, Japan) kit was used to synthesize cDNA according to the instruction manual. q-PCR for cDNA amplification was performed with Green™ Premix Ex Taq™ II (TaKaRa) kit. GAPDH was used as endogenous control and primers were shown in Supplementary Table S1. The expression of signature DE-FLs were normalized using the relative quantification method of 2-ΔΔCt.

### Statistical Analysis

Statistical procedures were applied using R v.4.0.3 and Prism v.8.0.0. The Student’s *t-test* or the Wilcoxon test was utilized for differences analysis. *p* < 0.05 was statistically significant.

## Results

### Screening of DE-FLs in CRC

**FIGURE 2:**
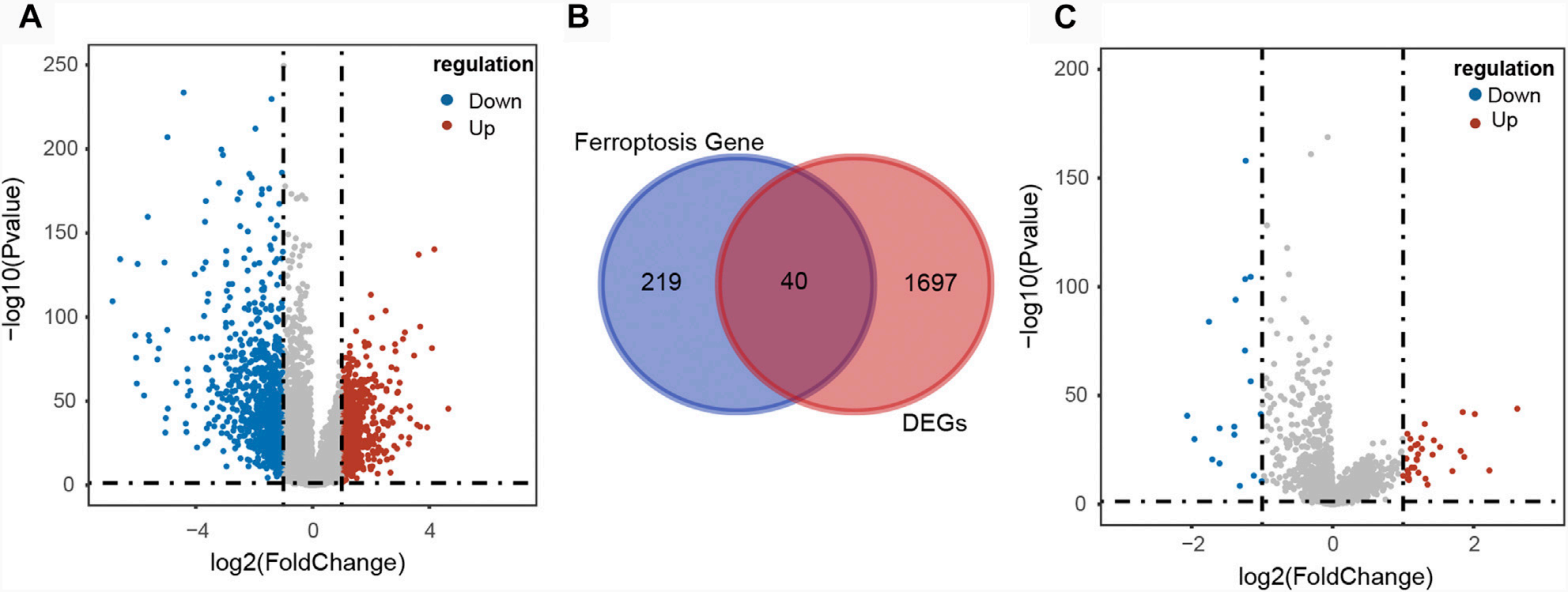
Identification of differentially expressed ferroptosis-related lncRNAs in CRC patients. **(A)** Volcano plot of the DEGs in CRC patients. **(B)** Venn diagram showed the intersection of 40 differentially expressed ferroptosis-related genes. **(C)** Volcano plot of the differentially expressed lncRNAs in CRC patients.

A total of 1737 DEGs, including 780 up-regulated and 957 down-regulated genes, between CRC and normal samples were obtained from TCGA (Figure 2A). And 382 FRGs were downloaded from the FerrDb database. Then 40 DE-FGs were identified by Venn diagram (Figure 2B) and the correlation among them was shown in Supplementary Figure S1. In addition, there were 51 DLRs selected in CRC, including 33 up-regulated and 18 down-regulated lncRNAs (Figure 2C). Finally, we performed Pearson correlation analysis to calculate the correlation between the DLRs and DE-FGs, and used |cor| > 0.3 and *p* < 0.05 as the selection criteria. As shown in Supplementary Data S1, 49 lncRNAs were acquired and termed as DE-FLs for further research.

### Construction of Ferroptosis-Related lncRNA Prognostic Signature in CRC

**FIGURE 3:**
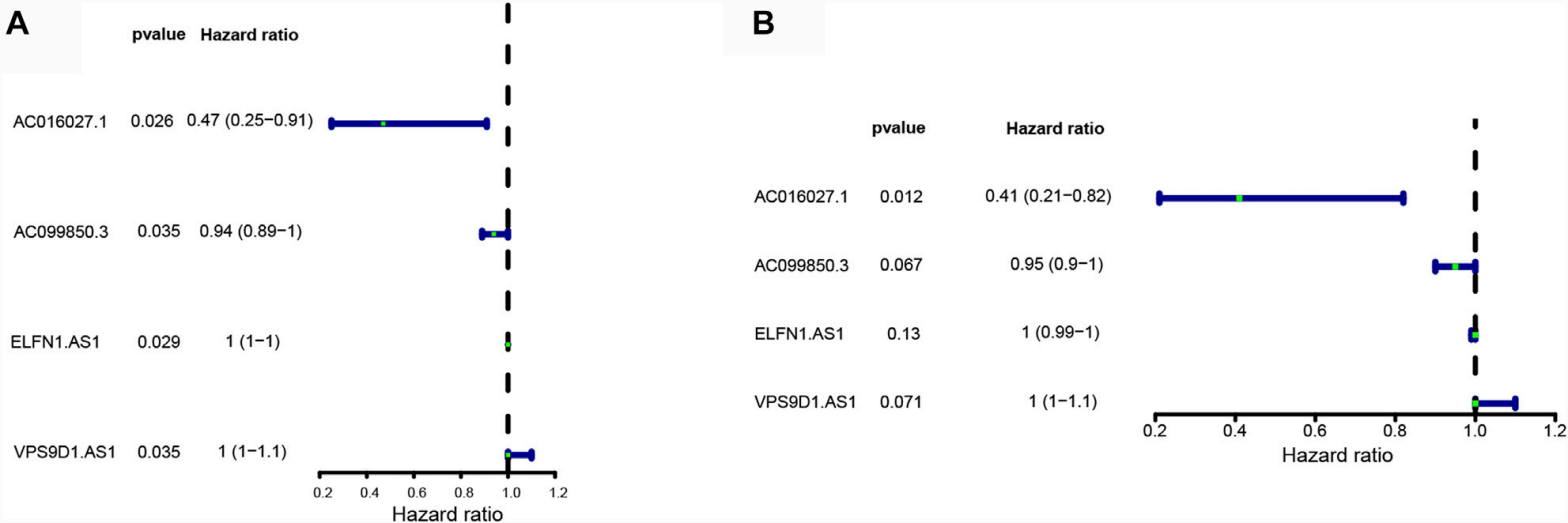
Construction of ferroptosis-related lncRNA prognostic signature in CRC patients. **(A)** Forest plots showed 4 DE-FLs selected by the univariate Cox regression. **(B)** Forest plots showed four prognostic DE-FLs identified by the multivariate Cox regression.

Univariate Cox regression analysis showed that 4 DE-FLs (AC016027.1, AC099850.3, ELFN1-AS1, and VPS9D1-AS1) were associated with OS in CRC, except VPS9D1-AS1 played a risk factor with HR > 1, the others acted as protectors with HR < 1 (Figure 3A). Multivariate Cox regression analysis further ascertained these 4 DE-FLs with prognostic significance (Figure 3B). Thus, they were employed to construct a prognostic signature model. Supplementary Figure S2 showed the survival probability of CRC patients in high- and low-expression DE-FLs respectively.

### Evaluation and Validation of the Ferroptosis-Related lncRNA Prognostic Signature

**FIGURE 4:**
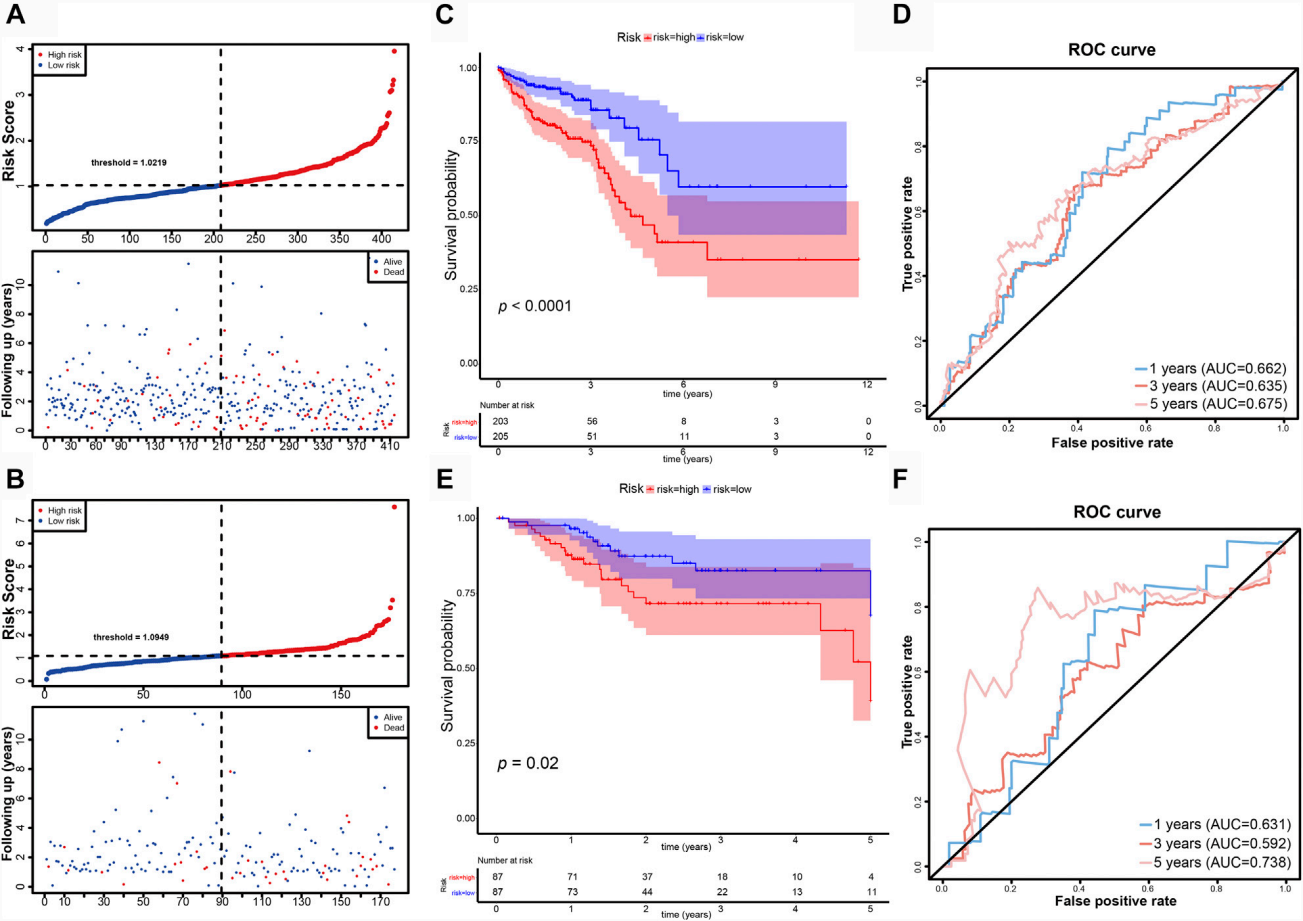
Evaluation and validation of the DE-FLs prognostic signature in CRC patients. **(A, B)** Distribution and Survival status of CRC patients with different risk scores in training set and validation set. **(C)** Kaplan–Meier survival curve of high- and low-risk group in training set. **(D)** The 1-, 3-, and 5-years ROC curve for risk model in training set. **(E)** Kaplan–Meier survival curve of high- and low-risk group in testing set. **(F)** The 1-, 3-, and 5-years ROC curve for risk model in testing set.

CRC patients in the TCGA dataset were classified into high-risk (training set n = 205; validation set n = 87; total n = 292) and low-risk (training set n = 203; validation set n = 87; total n = 290) groups using the median risk-score as the cutoff point (Figures 4A,B). Kaplan-Meier survival curve analysis showed that the OS of high-risk group patients were significantly poorer than low-risk group in the training set (n = 408) (Figure 4C). The 5-years survival rates were approximately 40 and 75% of the high-risk and low-risk group respectively. Time-dependent ROC curve analysis showed an appropriate accuracy of the prognostic signature in predicting OS in CRC, and AUC values were 0.662 at 1 year, 0.635 at 3 years, and 0.657 at 5 years (Figure 4D). For further validation, we confirmed that the results in the validation set coincided with the outcomes in the training set. In the validation set (n = 174), the significant prognostic value was *p* = 0.02 (Figure 4E) and AUC values for 1-, three- and 5- year OS were 0.631, 0.592, and 0.738, respectively (Figure 4F).

**FIGURE 5:**
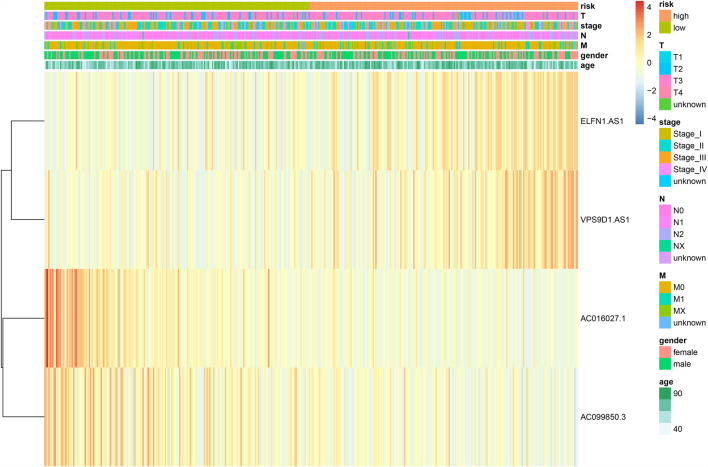
Relationship between the risk score and clinical significance in training set.

Then, we conducted the correlation analysis between the risk scores and the clinical characteristics of the CRC patients in TCGA database. We found that none of the clinical feature associated with risk scores in training set (Table 1 and Figure 5) and in validation set (Supplementary Table S2, Supplementary Figure S3).

**TABLE 1:** The relationship of CRC patients clinical feature and the DE-FLs model.

		Expression		
	Total (n = 408)	High (n = 205)	Low (n = 203)	*p*_value
Gender				
female	192 (47.1%)	99 (48.3%)	93 (45.8%)	0.687
male	216 (52.9%)	106 (51.7%)	110 (54.2%)	
Age (years)				0.138
≥ 60	290 (71.1%)	153 (74.6%)	137 (67.5%)	
<60	118 (28.9%)	52 (25.4%)	66 (32.5%)	
Pathologic stage				0.592
stage_Ⅰ	67 (16.4%)	32 (15.6%)	35 (17.2%)	
stage_Ⅱ	152 (37.3%)	74 (36.1%)	78 (38.4%)	
stage_Ⅲ	122 (29.9%)	61 (29.8%)	61 (30.0%)	
stage_Ⅳ	53 (13.0%)	32 (15.6%)	21 (10.3%)	
unknown	14 (3.4%)	6 (2.9%)	8 (3.8%)	
T stage				
T1	9 (2.2%)	6 (2.9%)	3 (1.5%)	0.568
T2	71 (17.4%)	32 (15.6%)	39 (19.2%)	
T3	290 (71.1%)	146 (71.2%)	144 (70.9%)	
T4	37 (9.1%)	20 (9.8%)	17 (8.4%)	
unknown	1 (0.2%)	1 (0.5%)	0 (0%)	
M stage				0.174
M0	302 (74.0%)	148 (72.2%)	154 (75.9%)	
M1	52 (12.7%)	32 (15.6%)	20 (9.9%)	
MX	45 (11.0%)	19 (9.3%)	26 (12.8%)	
unknown	9 (2.2%)	6 (2.9%)	3 (1.5%)	
N stage				0.285
N0	231 (56.6%)	112 (54.6%)	119 (58.6%)	
N1	92 (22.5%)	42 (20.5%)	50 (24.6%)	
N2	82 (20.1%)	49 (23.9%)	33 (16.3%)	
NX	2 (0.5%)	1 (0.5%)	1 (0.5%)	
unknown	1 (0.2%)	1 (0.5%)	0 (0%)	

### The Risk Score Is an Independent Prognostic Factor in CRC

**FIGURE 6:**
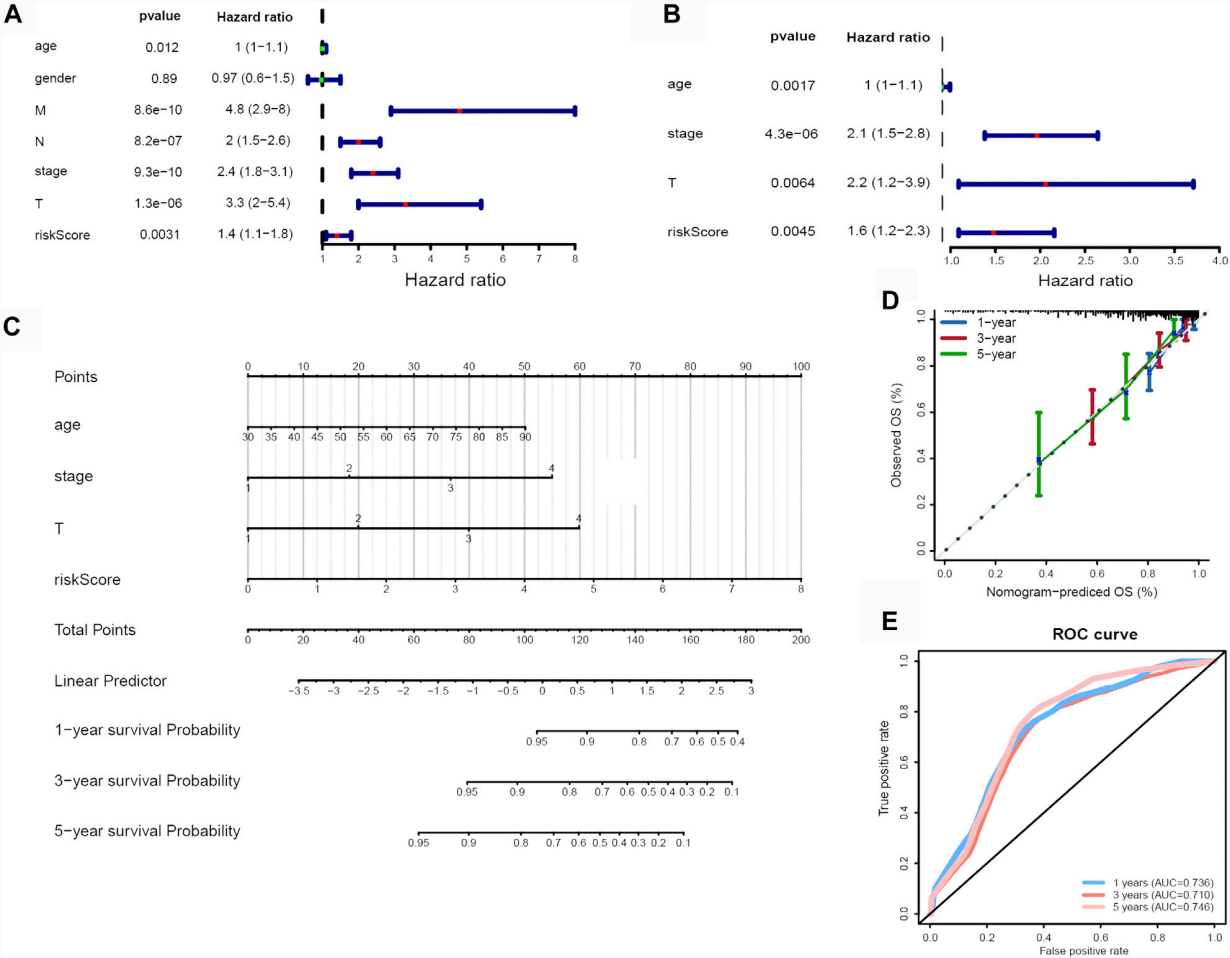
Identification of independent risk factors and construction of a nomogram. **(A)** The univariate and **(B)** multivariate Cox regression analysis of risk score and clinical feature prognostic value. **(C)** A nomogram was constructed to predict 1-, 3-, and 5-years OS using risk score, stage, T and age. **(D)** Calibration curves of nomogram. **(E)** 1-, 3-, and 5-years ROC curve based on nomogram.

In order to determine if the ferroptosis-related lncRNA prognostic signature was an independent prognostic factor for CRC patients, we performed univariate and multivariate Cox regression analyses. Univariate analyses showed that age, MNT (*p* < 0.001), stage (*p* < 0.001) and the risk score were significantly associated with OS (Figure 6A). Multivariate analyses showed that pathologic stage (*p* < 0.001), age, T stage and risk score could act as independent prognostic factor (Figure 6B).

Subsequently, we developed a nomogram to predict 1-, 3-, and 5-years OS of CRC using the all of the independent prognostic factors (Figure 6C). The C-index for the nomogram was 0.789. The 1-year, 3-years and 5-years calibration curves showed the nomogram with an accurate prediction in CRC (Figure 6D). Finally, the AUC values at 1-year, 3-years and 5-years were 0.736, 0.710, 0.746, respectively (Figure 6E), also indicated the predictive capacity of the nomogram was reliable.

### Functional Analysis of Ferropotosis-Related lncRNA Signature

**FIGURE 7:**
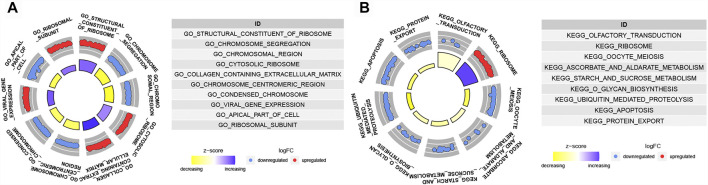
Functional enrichment analysis. **(A)** GO items enriched by GSEA analysis in high risk group. **(B)** KEGG pathways enriched by GSEA analysis in high risk group.

GSEA analyses were conducted to further explore the difference biological mechanism between low- and high-risk groups. In high-risk group, the biological process mainly related to chromosome (Figure 7A). And the pathways such as OLFACTORY_TRANSDUCTION, OOCYTE_MEIOSIS, ASCORBATE_AND_ ALDARATE_METABOLISM, O_GLYCAN_BIOSYNTHESIS, STARCH_AND_ SUCROSE_METABOLISM, UBIQUITIN_MEDIATED_PROTEOLYSIS, APOPTOSIS and PROTEIN_EXPORT were significantly enriched in the high-risk group (Figure 7B). Except RIBOSOME, all of the others were down-regulated.

### Construction of Co-expression Network

**FIGURE 8:**
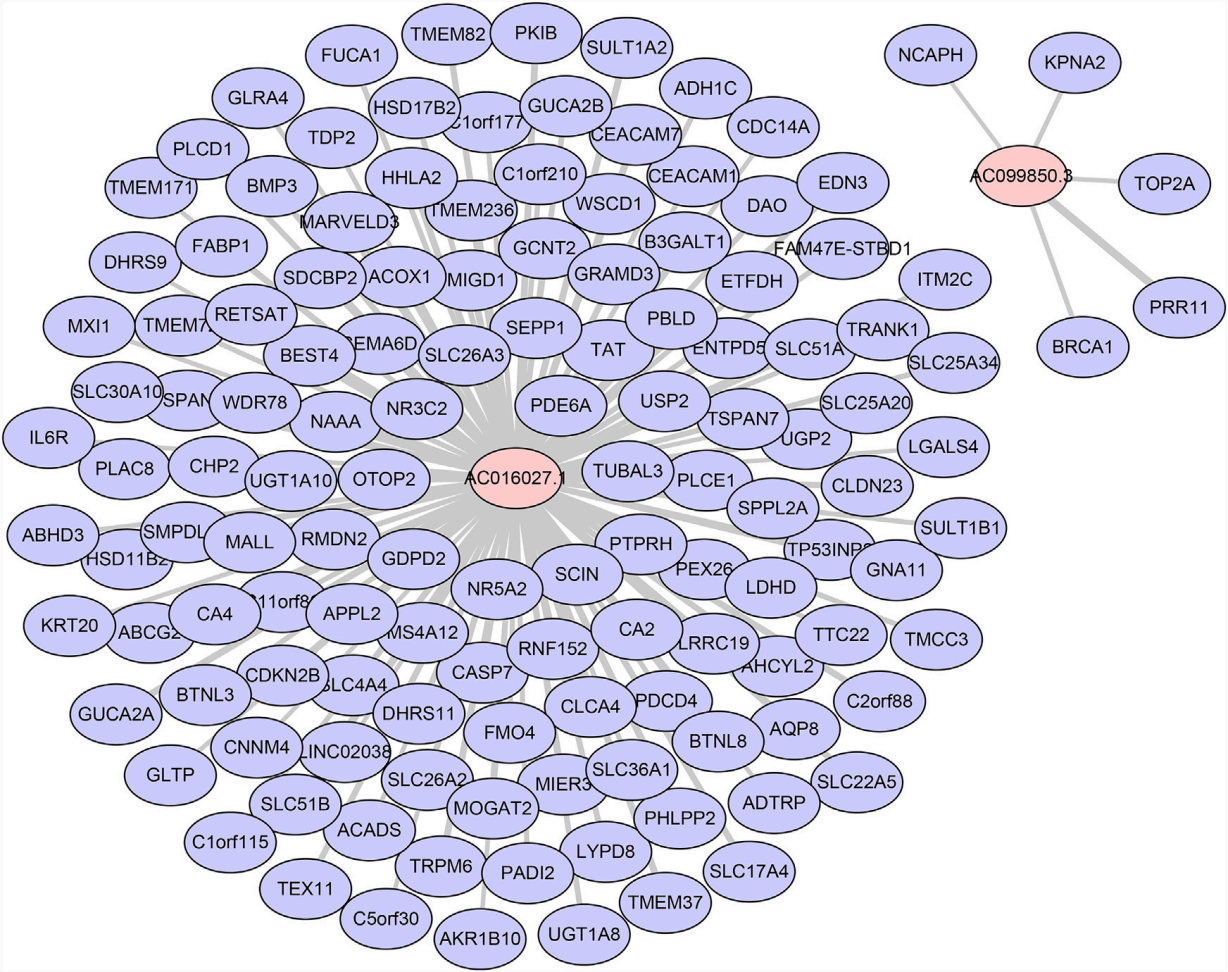
Co-expression network of DE-FLs and mRNA.

We used Pearson correlation analyses and Cytoscape to construct lncRNA-mRNA co-expression network. When threshold parameter |cor| > 0.7 was set, 132 mRNA which significantly related to two prognostic DE-FLs (AC016027.1, AC099850.3) were involved in the network (Figure 8, Supplementary Data S2). Pearson correlation analysis also showed all the mRNAs (|cor| > 0.5) associated with these 4 DE-FLs (Supplementary Data S3). PEX26, SLC51B, TMEM236, CA4, and SLC26A3 ranked as top five genes high correlated with AC016027.1. PRR11, BRCA1, KPNA2, TOP2A and NCAPH were top five high correlated with AC099850.3.

### Functional Enrichment Analysis

**FIGURE 9:**
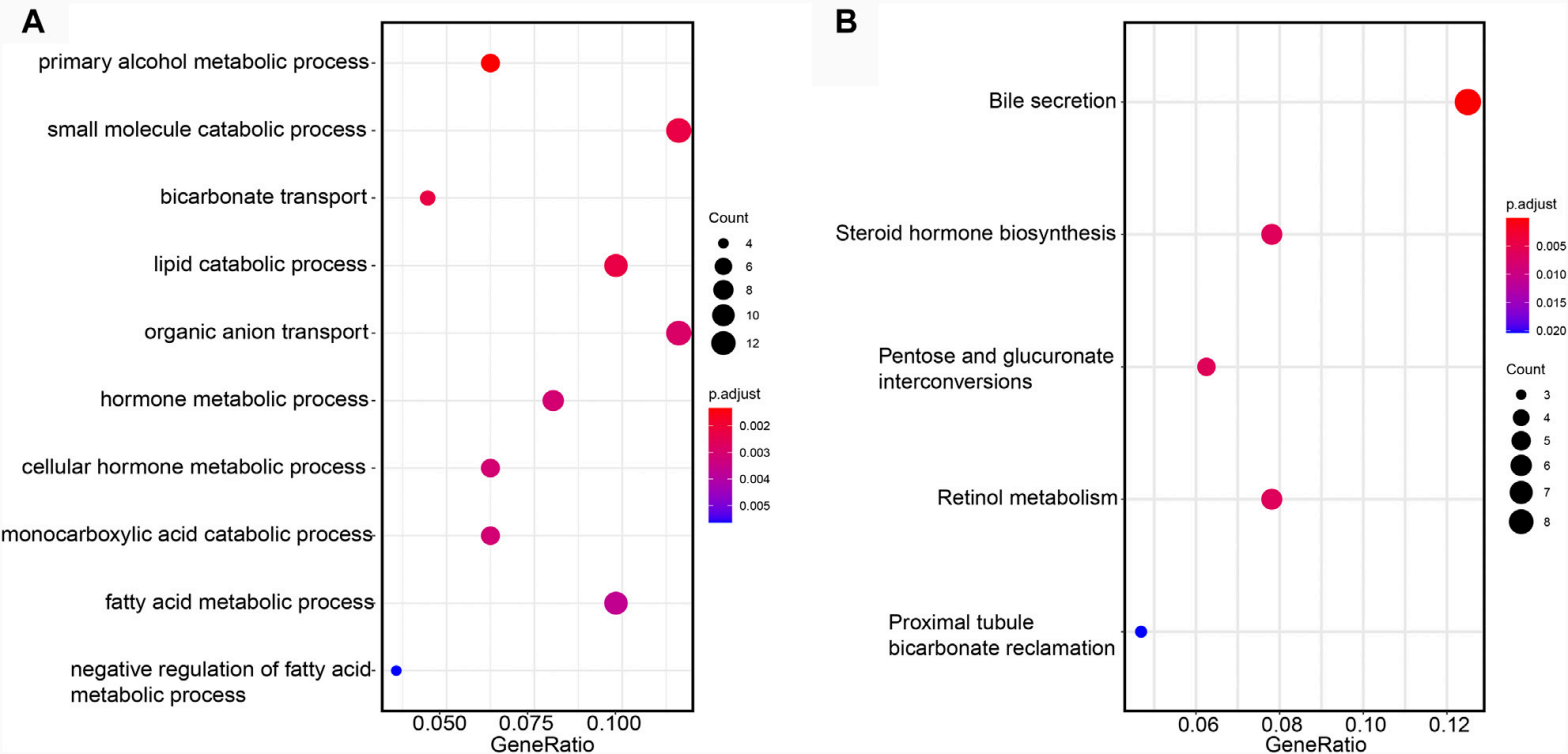
Functional annotation of mRNA. **(A)** GO items enriched by GSEA analysis on the network-genes. **(B)** KEGG pathways enriched by GSEA analysis on the network-genes.

To investigate the biological pathways regulated by the prognostic lncRNAs, we performed GO and KEGG enrichment analysis on the network-genes. Five KEGG pathways and 84 GO functional items were enriched. The top ten GO items were mainly related with small molecule catabolic process, lipid catabolic process, fatty acid metabolism process (Figure 9A). KEGG pathway analysis confirmed that bile secretion was the most significantly enriched pathway (Figure 9B).

### Validation of the Ferrptosis-Related Signature in Clinical CRC Tissues

**FIGURE 10:**
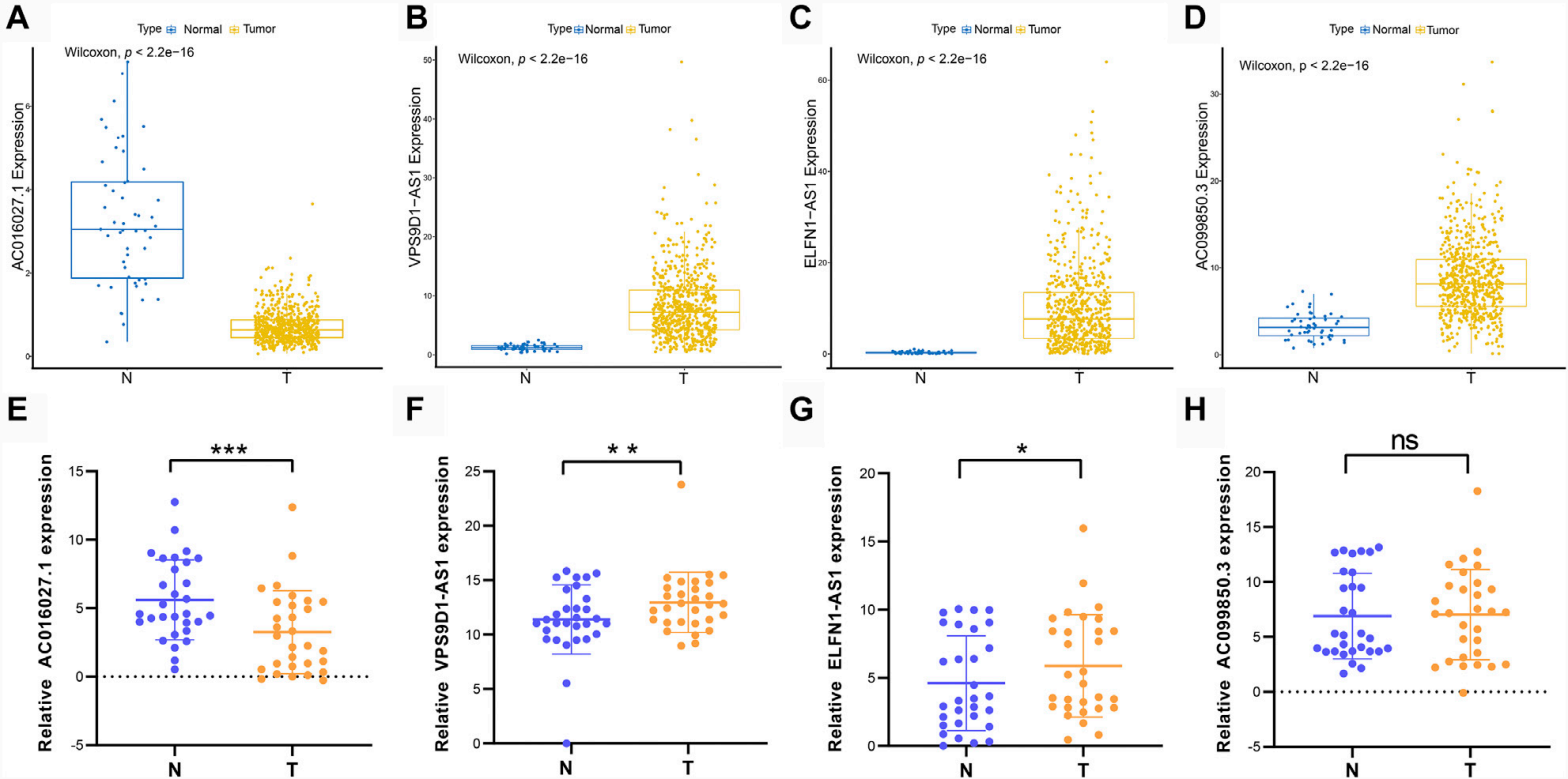
Different expressions of prognostic DE-FLs in CRC tissues and TCGA. **(A–D)** The different expression levels of 4 DE-FLs in TCGA. **(E–G)** Q-PCR results demonstrated the down-regulated expression level of AC016027.1 and up-regulated level of VPS9D1-AS1 and ELFN1-AS1 in CRC tissues compared with paired normal tissues. **(H)** Q-PCR result of expression level of AC099850.3 in CRC tissues compared with paired normal tissues. *, *p* < 0.05. **, *p* < 0.01. ***, *p* < 0.001.

The expression levels of prognostic DE-FLs were detected by q-PCR in thirty pairs of CRC tissues and adjacent tissues. As shown in Figures 10A–D, AC016027.1 was down-regulated in CRC from TCGA database whereas VPS9D1-AS1, ELFN1-AS1 and AC099850.3 were up-regulated. Q-PCR analyses showed that expression level of AC016027.1 was down-regulated in CRC tissues compared with paired normal tissues; VPS9D1-AS1 and ELFN1-AS1 were up-regulated (Figures 10E–G) which were consistent with the results from TCGA. There was no significant difference in the expression level of AC099850.3 between CRC and normal tissues (Figure 10H).

## Discussion

A growing body of researches showed that aberrant expression of lncRNAs were associated with risk for CRC (Ni et al., 2019; Wang Y. et al., 2019; Yuan et al., 2021). Their results indicated the clinic potential of lncRNAs as stratification markers, diagnostic indicators, therapeutic targets for CRC (Lin and Yang, 2018). Ferroptosis, dependent on iron and reactive oxygen species (ROS) and characterized by lipid peroxidation, is a non-apoptotic regulated cell death (Kajarabille and Latunde-Dada, 2019). Much evidence has indicated that ferroptosis participated in multiple pathways and was critical for eradicating the carcinogenic cells (Dixon, 2017; Mou et al., 2019). Xu et al. (2020) demonstrated that knockout of SLC7A11 induced ferroptosis, thereby suppressed the progression of CRC stem cells. Previous studies which investigated the function of ferroptosis in CRC have largely concentrated on TP53 (Murphy, 2016; Xie et al., 2017). Xie et al. (2017) reported that TP53 suppressed ferroptosis triggered by erastin through the inhibition of dipeptidyl-peptidase-4 (DPP4) in a transcription-independent manner in CRC. Emerging evidences indicated that lncRNAs have been involved in tumorigenesis and tumor progression by targeting ferroptosis-related genes, such as p53RRA which restrains ferroptosis-modulated genes in a p53-dependent manner by interacting with G3BP1 (Mao et al., 2018). Mao C *et al.* validated a novel mechanism that p53RRA regulates apoptosis and ferroptosis in cancer. Also, p53RRA can be used as a prognostic marker in lung adenocarcinoma patients. Therefore, particular emphasis should be placed on ferroptosis-related lncRNAs for individualized diagnosis and treatment in CRC patients. In present study, we screened four ferroptosis-related lncRNAs and constructed a ferroptosis-related risk model which might be a potential diagnostic biomarker, using bioinformatics and statistical tools.

Firstly, we examined 49 DE-FLs in CRC by analyzing TCGA-COADREAD data set (Smyth, 2004), then 4 DE-FLs (AC016027.1, AC099850.3, ELFN1-AS1, and VPS9D1-AS1) that significantly correlated with OS were found to construct the risk signature according to the univariate and multivariate Cox regression analysis. CRC patients in high-risk groups showed shorter OS compared to those in low-risk groups. Kaplan-Meier survival curve and ROC curve evaluated the predictive accuracy of the ferroptosis-related signature in CRC patients (Robin et al., 2011).

Previous research reported that AC099850.3 and ELFN1-AS1 participated in prognostic autophagy-related lncRNAs signature in HCG patients (Jia et al., 2020). Jiang Q. et al. (2021) reported AC099850.3 was selected by a prognostic autophagy-related lncRNAs signature in oral and oropharyngeal squamous cell carcinoma. The above results appeared to support an existing viewpoint that ferroptosis is a type of autophagy-dependent cell death because a lot of autophagy-related signal pathways contributed to ferroptosis, including BECN1-mediated system xc-inhibition and NCOA4-facilitated ferritinophagy (Song et al., 2018; Quiles Del Rey and Mancias, 2019; Zhou et al., 2020). ELFN1-AS1 was validated with high expression in colon cancer tissues and cells and was reported to promote proliferation and invasion of colon cancer cells by adjusting the miR-191-5p/SATB1 axis (Du et al., 2020). Liu et al. (2020) suggested that VPS9D1-AS1 was a competing endogenous RNA in CRC cells and increased the expression of HMGA1, thereby influenced CRC progression. However, to our knowledge, AC016027.1 and AC099850.3 have not been reported in CRC, which means our findings indicated further research is necessary.

In addition, the ferroptosis-related lncRNA signature is an independent prognostic factor. We constructed a robust nomogram integrating risk score and prognostic clinical features including age, pathological staging and T stage for predicting patient outcomes. ROC curve further demonstrated that this nomogram provided a personalized and accurate survival prediction. Collectively, in our study, there are plenty of evidences suggested that the ferroptosis-related lncRNA signature made accurately predictive prognosis of CRC patients and showed great potential for clinical individualized prognosis and therapy.

Ferroptosis-related GO terms and KEGG signaling pathways were enriched, in order to illustrate the specific mechanism behind the predictive signature. We identified APOPTOSIS pathway reported to be closely associated with ferroptosis and the expression levels of genes involved in the pathway were significantly upregulated in the high-risk group. Previous study revealed an existing cross talk between ferroptosis and apoptosis through ferroptosis-induced endoplasmic reticulum stress (Lee et al., 2018). C/EBP homologous protein (CHOP) signaling pathway-mediated p53 upregulated modulator of apoptosis (PUMA) expression participated in the cooperative interaction between ferroptosis and apoptosis, indicating combination of ferroptotic and apoptotic agent treatment could be considered as a new therapeutic strategy for cancer. Meanwhile, the interaction network between prognostic lncRNAs and DE-FGs was also constructed. The correlation analysis gave us clues about the regulatory relationship between these lncRNAs and mRNA, as well as the molecular mechanism of their role in colorectal cancer. Through the above analysis, we preliminarily inferred that these four ferroptosis-related lncRNAs may directly or indirectly regulate DE-FGs or genes participated in the pathways which were closely related to CRC and thus caused the difference in patient survival.

Nie et al. (2021) comprehensively reported construction of a ferroptosis related genes prognosis model in colon cancer, which aimed to predict survival probability of patients. Our study further screened for differently expressed lncRNAs in CRC which were associated with ferroptosis-related genes, and then constructed a ferroptosis-related lncRNA prognosis model of CRC by Cox regression analyses. There are some advantages in our study that not only did we conduct the mining and exploration of public databases, but also developed the biochemical experiment such as q-PCR to verify our findings in clinical CRC patient samples. However, the shortcoming is that the specific biological molecular mechanisms of these ferroptosis-related prognostic lncRNAs have not been studied in depth. Therefore, future studies are required to explore its exact molecular functions of these ferroptosis-related prognostic genes in CRC.

In conclusion, our study constructed and validated a ferroptosis-related lncRNA prognosis signature in CRC, which consist of AC016027.1, AC099850.3, ELFN1-AS1 and VPS9D1-AS1. The novel ferroptosis-related lncRNA prognosis signature accurately predicts the survival of CRC patients and differentiates them into high- and low-risk groups. Furthermore, the prediction model was independent of clinical features and a reliable nomogram was constructed. Our results might shed lights on promising biomarkers and targets for the individualized therapy of CRC.

## Data Availability

The three transcriptome datasets used in this study are all publicly available. The datasets TCGA-COAD and TCGA-READ for this study can be found in the National Cancer Institute GDC Data Portal [https://portal.gdc.cancer.gov]. The gene set FRGs was downloaded from the FerrDb database [http://www.zhounan.org/ferrdb]. All other data generated in this study are included in the article or the Supplementary Material.
