# Supplementary material for: Construction and Validation of a Novel Ferroptosis-Related lncRNA Signature to Predict Prognosis in Colorectal Cancer Patients
Source: Front Genet. 2021 Oct 28;12:709329. doi: 10.3389/fgene.2021.709329 (PMC8581609; doi:10.3389/fgene.2021.709329)
Supplement: Supplementary file 3 [file DataSheet1.pdf]

Supplementary Material

1 Supplementary Figures and Tables

1.1 Supplementary Figures

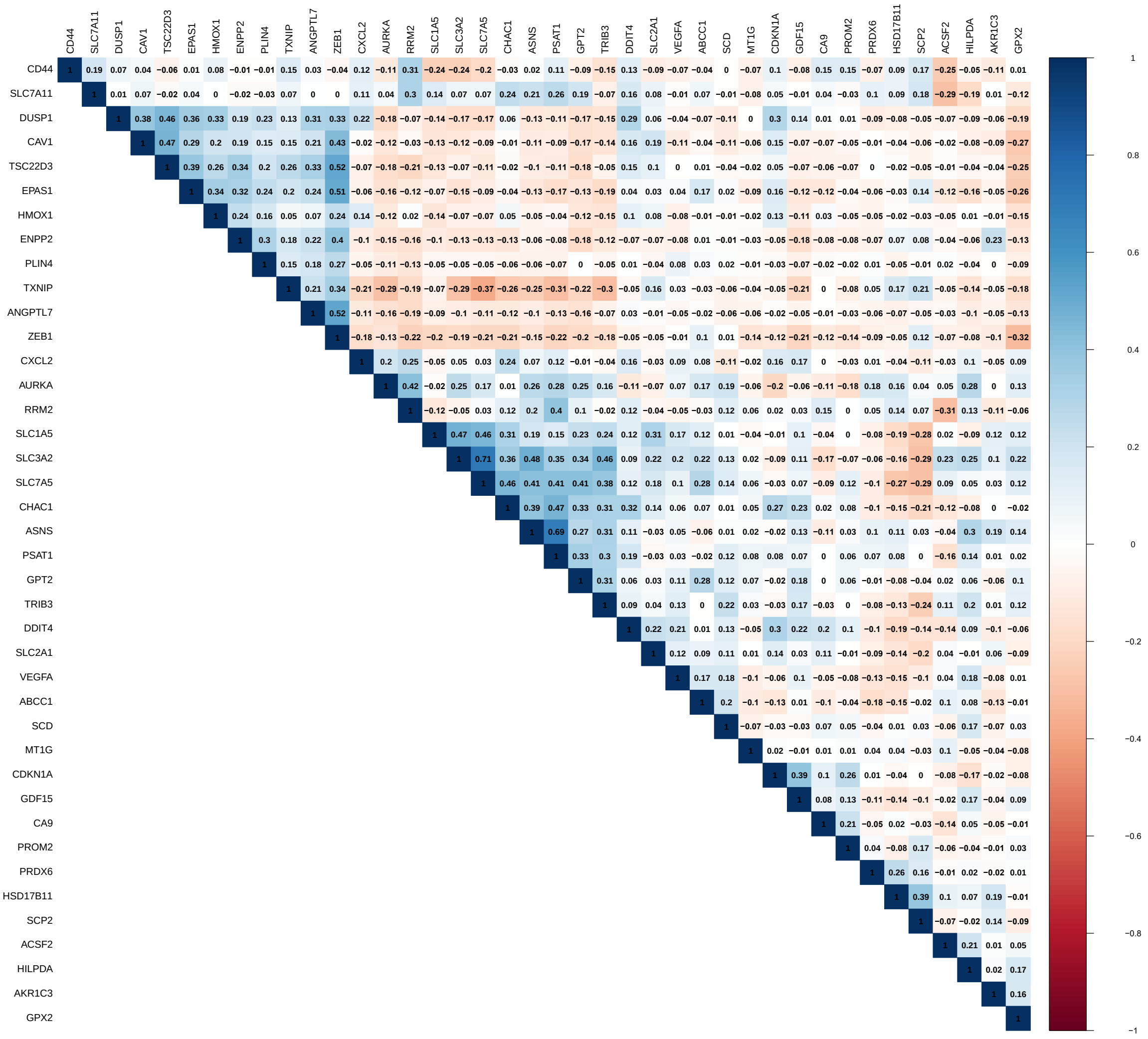

Supplementary Figure 1. The correlation between DE-FGs in CRC. The larger the correlation coefficient, the darker the color. Red is positively correlated and blue is negatively correlated.

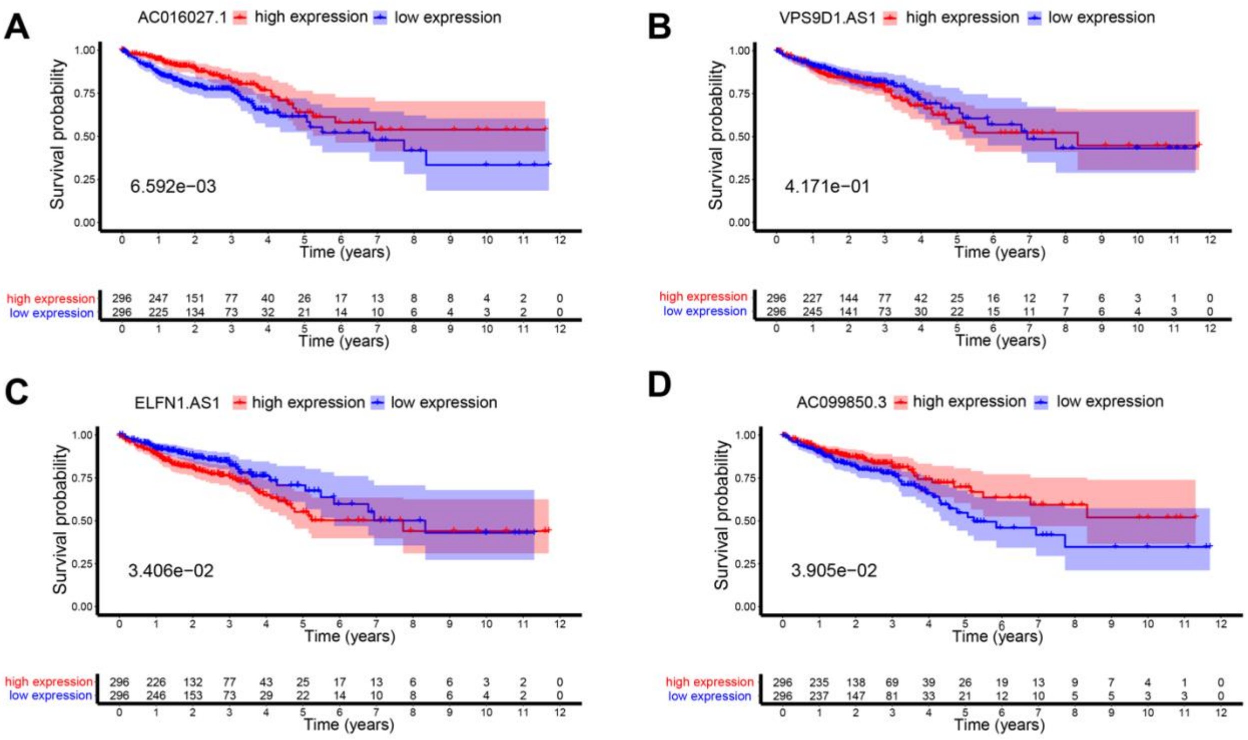

Supplementary Figure 2. CRC patient survival probabilities in TCGA database. (A-D) Kaplan–Meier survival curve of each high- and low- prognostic DE-FLs expression groups.

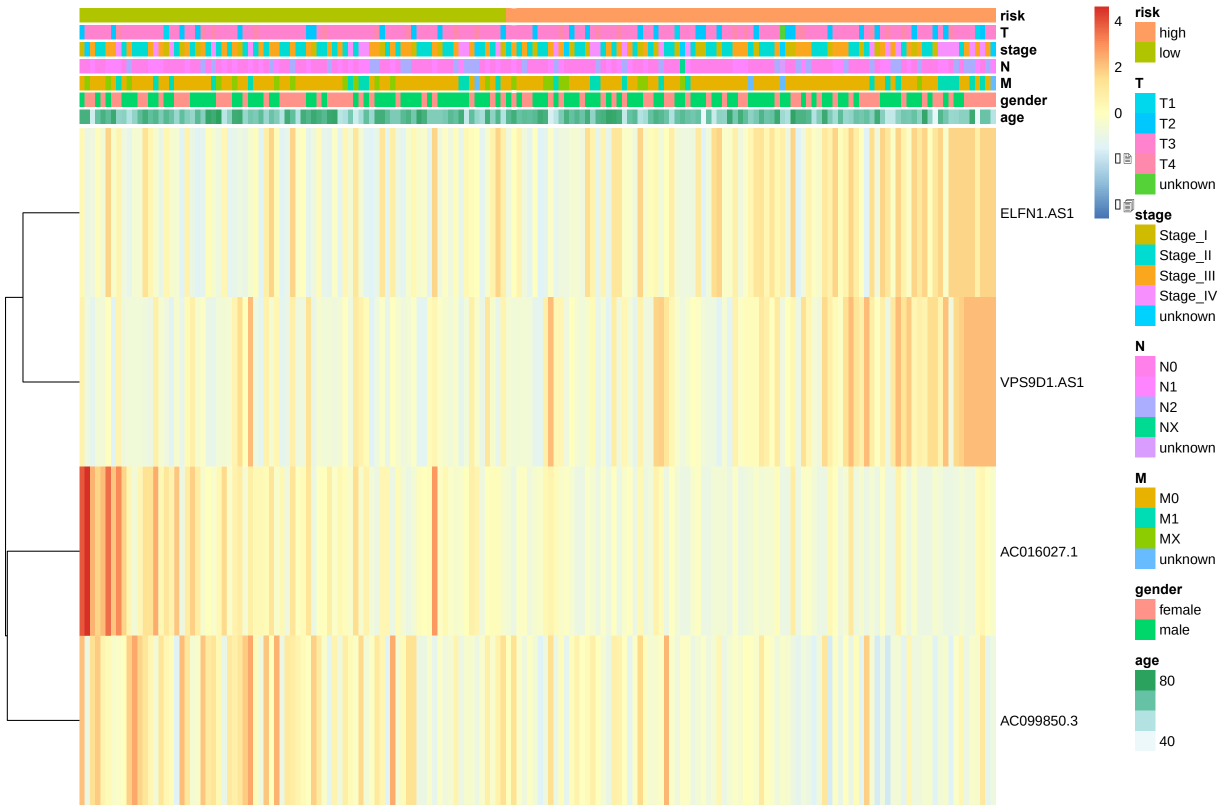

Supplementary Figure 3. Relationship between the risk score and clinical significance in validation set.

1.2 Supplementary Table

Supplementary Table 1. Sequences of primers used in q-PCR.

		Primers: 5'- 3'
AC016027.1	F:<br>R:	CCCATCGCCTTCTGTAATGGA<br>TGCTTCGTTTTGACTTCCGC
VPS9D1-AS1	F:<br>R:	AGATCCACAATGCCGTAGAC<br>CTTGGAGGCAGCTGTGTTTAG
ELFN1-AS1	F:<br>R:	AAAAGTTGACGCCGCATTCT<br>GAGAATGGATTGTGGGTGCC
AC099850.3	F:<br>R:	CGTCTTTCACCCAGCCTCTT<br>AAAGCAGGAACCCCTCTGTG
GAPDH	F:<br>R:	TCGGAGTCAACGGATTTGGTCGT<br>TGCCATGGGTGGAATCATATTGGA

Supplementary Table 2. The relationship of CRC patients clinical feature and the DE-FLs model in validation set.

	Total	Expression		*p*_value
	(n=174)	High (n=92)	Low (n=82)	
Gender				
female	75 (43.1 %)	40 (43.5 %)	35 (42.7 %)	1
male	99 (56.9 %)	52 (56.5 %)	47 (57.3 %)	
Age (years)				1
>= 60	131 (75.3 %)	69 (75.0 %)	62 (75.6 %)	
< 60	43 (24.7 %)	23 (25.0 %)	20 (24.4 %)	
Pathologic stage				0.798
stage_Ⅰ	27 (15.5 %)	16 (17.4 %)	11(13.4 %)	
stage_Ⅱ	64 (36.8 %)	32 (34.8 %)	32 (39.0 %)	
stage_Ⅲ	51 (29.3 %)	25 (27.2 %)	26 (31.7 %)	
stage_Ⅳ	26 (14.9 %)	15 (16.3 %)	11 (13.4 %)	
unknown	6 (3.4 %)	4 (4.3 %)	2 (2.4 %)	
T stage				
T1	3 (1.7 %)	2 (2.2 %)	1 (1.2%)	0.766
T2	29 (16.7 %)	17 (18.5 %)	12 (14.6 %)	
T3	121(69.5 %)	61 (66.3 %)	60 (73.2 %)	
T4	20 (11.5 %)	11 (12.0 %)	9 (11%)	
unknown	1 (0.6 %)	1 (1.1 %)	0 (0 %)	
M stage				0.599
M0	123 (70.7 %)	65 (70.7 %)	58 (70.7 %)	
M1	26 (14.9 %)	15 (16.3%)	11 (13.4 %)	
MX	21 (12.1 %)	9 (9.8%)	12 (14.6 %)	
unknown	4 (2.3 %)	3 (3.3 %)	1 (1.2 %)	
N stage				0.677
N0	97 (55.7 %)	52 (56.5 %)	45 (54.9 %)	
N1	44 (25.3 %)	21 (22.8 %)	23 (28.0 %)	
N2	31 (17.8 %)	17 (18.5 %)	14 (17.1 %)	
NX	1 (0.6 %)	1 (1.1 %)	0 (0 %)	
unknown	1 (0.6 %)	1 (1.1 %)	0 (0 %)	
